# Is faster always better? A critical assessment of *Vibrio natriegens* as microbial host for recombinant protein production

**DOI:** 10.1007/s00449-026-03352-4

**Published:** 2026-05-27

**Authors:** Stefanie Brückner, Maike Scherer, Matthias Tobler, Bastian Blombach, Kathrin Castiglione

**Affiliations:** 1 Institute of Bioprocess Engineering, Friedrich-Alexander-Universität Erlangen-Nürnberg, Paul-Gordan-Straße 3, 91052 Erlangen, Germany; 2 Microbial Biotechnology, Campus Straubing for Biotechnology and Sustainability, Technical University of Munich, 94315 Straubing, Germany; 3 SynBiofoundry@TUM, Technical University of Munich, 94315 Straubing, Germany

**Keywords:** *Vibrio natriegens*, Vmax X2, *Escherichia coli*, Gene expression, Membrane proteins, Outer membrane porins, Salt requirement, Genetic tools, Codon usage, Autoinduction, Scale-up

## Abstract

The marine bacterium *Vibrio natriegens* has emerged as a promising candidate for recombinant protein production, primarily due to its rapid growth rate combined with its high protein synthesis capacity. These attributes have the potential to shorten production processes while achieving high yields of heterologously produced proteins. This review provides a concise overview of the substantial progress that has been made in the development of genetic tools and expression systems for *V. natriegens*, highlighting their successful application to the production of a wide range of proteins, including challenging targets such as membrane proteins. In addition, it critically examines the advantages and limitations of this organism as a production host, with a particular focus on whether its rapid growth translates into a tangible benefit for recombinant protein production. By contrasting the current status of *V. natriegens* with established *Escherichia coli*-based systems, key technological and biological developments required for its broader adoption are identified. In particular, the scale-up of protein production for potential industrial implementation remains underexplored and may present significant challenges due to the physiological demands of *V. natriegens*, including high oxygen requirements and the need for salt-supplemented media. Ultimately, whether fast growth equates to superior productivity, and whether *V. natriegens* can establish itself as a robust and broadly applicable protein production host, will depend on continued strain development and process intensification.

## Introduction

The marine bacterium *Vibrio natriegens* (*V. natriegens*) was first isolated from salt marsh mud on Sapelo Island, Georgia, in 1958 and was soon recognized for its exceptionally rapid growth with a doubling time of less than ten minutes [1, 2]. However, in the following decades, this organism received little scientific attention and was mainly used for educational purposes due to its fast growth [3, 4].

The interest in *V. natriegens* as a potential platform organism increased noticeably after 2016, when Weinstock *et al.* developed genetic tools and methods as well as engineered strains that improved its suitability for biotechnological applications [5, 6]. Since then, an increasing number of studies has investigated the physiology, genome and metabolism of *V. natriegens* [7, 8]. Advances in genome sequencing and the continuous expansion of its molecular biology toolbox [9–13] along with the adaption of genetic methods and vectors originally developed for *Escherichia coli* (*E. coli*) [14–16], have further enabled its use in metabolic engineering campaigns and the production of diverse chemical compounds [14, 17–20]. Recently, *V. natriegens* has gained attention as an alternative to *E. coli* as a potential protein production host [5, 6, 16, 21] and has already been used for the recombinant production of diverse proteins [5, 6, 16, 21–25]. A very recent review has provided a comprehensive overview of *V. natriegens* as a protein production chassis, covering physiological properties, genetic tools and expression strategies [26]. However, despite the increasing interest, the suitability of *V. natriegens* as a production host remains less well characterized compared to the standard chassis *E. coli*. In particular, key aspects relevant for biotechnological application, such as quantitative productivity metrics, process related limitations including oxygen supply and scale-up as well as the production of difficult targets, remain only marginally addressed, especially in the context of comparisons to well-established and optimized *E. coli* production strains and strategies. This review therefore aims to critically reflect on the current state of development of *V. natriegens* as novel protein production host and highlight both advantages and actual bottlenecks in comparison to *E. coli*. In this context, beside its fast growth, important factors such as its genetic accessibility, ease of cultivation and overall suitability for recombinant protein production, particularly for challenging targets like membrane proteins, will be evaluated.

## General characteristics of *V*. *natriegens* in comparison to *E. coli*

*V. natriegens* is a non-pathogenic, Gram-negative, facultative anaerobic member of the Gammaproteobacteria [27–29]. After isolation, the organism was initially classified within the genus *Pseudomonas* and later reassigned to the genus *Vibrio* in the 1970s based on *Vibrio*-typical morphological and phenotypical characteristics, supported by a guanine-cytosine (GC) content of 45%, which deviated from the high GC-ratios characteristic of *Pseuodomonas* species [28, 30]. The strain ATCC 14048 (DSM 759) represents the commonly used wildtype strain and is used in most physiological and genetic studies [27]. The genome of *V. natriegens* ATCC 14048 has been fully sequenced, revealing a total size of 5.2 Mb, organized into two chromosomes, with the majority of essential genes located on chromosome I [9–11]. In contrast, the genome of *E. coli* BL21 (DE3) comprises only a single chromosome with a total size of 4.6 Mb. Comparative genomic analysis of *E. coli* and *V. natriegens* showed that, despite their overall similarity, *V. natriegens* exhibits an enrichment of ribosomal- and translation-related genes as well as a higher number of rRNA operons. Compared to *E. coli*’s 7 rRNA operons, there are a least 13 rRNA operons shown for *V. natriegens* [31]. In addition, *V. natriegens* harbors strong promoters, reflecting its capacity for rapid growth and high protein synthesis rates. Notably, a comparison of the rRNA P1 und P2 promoters of *V. natriegens* with *E. coli*’s P1 und P2 promoters revealed a 16% higher promoter activity [9, 31]. Moreover, substantially accelerated growth is observed for *V. natriegens* compared to *E. coli*, reaching growth rates of up to 4.43 h^− 1^ in complex medium and 1.97 h^− 1^ in minimal medium with glucose [1, 32] (Table 1). The resulting extremely short doubling times of up to less than 10 min represent one of the most distinctive features of *V. natriegens* and has been highlighted as advantage for biotechnological applications [6, 33].

However, in contrast to *E. coli*, which grows efficient in low-salt media, *V. natriegens* is moderately halophilic [28]. Therefore, optimal cultivation conditions resemble those of *E. coli* with temperatures between 30 and 37 °C and slightly alkaline pH. However, the media must be supplemented with sodium chloride (NaCl) or sea salts [32] (Table 1). Although *V. natriegens* tolerates a broad range of salinities, growth is severely reduced under low-salt conditions, limiting its direct use in standard *E. coli* media [34]. Moreover, the requirement for supplemented media with NaCl concentrations between 7.5 and 30 g L^− 1^ can complicate downstream processing and increase the risk of corrosion in bioreactor systems [32, 34, 35]. It has been demonstrated that elevated salt concentrations in the upstream medium can exert a disadvantageous influence on downstream processing by impairing solid-liquid separation [36] and altering chromatographic performance through modulation of protein-ligand interactions and reducing binding capacity [37, 38]. Moreover, elevated ionic strength can promote protein aggregation or precipitation, particularly during buffer exchange or dilution steps, thereby impacting yield and product quality [36]. In addition, increased salt levels may intensify membrane fouling during filtration steps, reducing the process efficiency and robustness [39]. Since sodium but not chloride is essential for growth of *V. natriegens*, Biener *et al.* [40] adjusted the media receipt and replaced NaCl by Na_2_HPO_4_, Na_2_SO_4_, and sodium citrate, which maintained the high growth rates. *V. natriegens* can use a variety of carbon sources, including sugars like glucose, galactose, maltose, arabinose, fructose, glycerol and certain organic acids [5, 34] (Table 1). However, in contrast to *E. coli*, *V. natriegens* is unable to metabolize lactose, as there is currently no evidence of genes responsible for lactose import and catabolism (*lacY* and *lacZ*) in *Vibrio* species [22, 41, 42]. Unfortunately, this prevents the use of lactose-based autoinduction systems that are widely used for recombinant protein production [43]. Notably, *V. natriegens* exhibits specific substrate consumption rates (q_s_) up to twice as high as those of traditional production hosts, such as *E. coli* and *Bacillus subtilis* (*B. subtilis)*, reaching 3.9 g g^− 1^ h^− 1^ under aerobic and 7.8 g g^− 1^ h^− 1^ for *V. natriegens* under anaerobic conditions, which provides an excellent basis for reaching high productivities in biotechnological processes [34]. Early bioprocess development studies have demonstrated successful scale-up for metabolite production using *V. natriegens*, revealing its potential for high cell-density cultivation, rapid substrate conversion and robust productivity [44, 45]. At laboratory-scale, biomass concentrations of up to 60 g cell dry weight per liter could be achieved with glucose-feeding approaches in mineral salt media [40, 44]. However, despite its broad carbon source flexibility and ability to grow in both complex and minimal media, the dependence on salt-supplemented media combined with the inability to utilize lactose requires the adaption of established *E. coli* cultivation strategies when using *V. natriegens* as a production host.

**Table 1:** Comparison of the most important features for the microbial hosts *V. natriegens* and *E. coli* regarding cultivation and recombinant protein production

	*V. natriegens*	*E. coli*
Oxygen requirement	Facultatively anaerobic [1]	Facultatively anaerobic [46]
Growth rate (aerobic)	Complex medium: 2.34 h^− 1^ to 4.43 h^− 1^ [1, 5, 32, 34] Minimal medium with glucose: 1.66 h^− 1^ to 1.97 h^− 1^ [32, 34]	Complex medium: up to 2 h^− 1^ (strain dependent, 1.53 h^− 1^ for *E. coli* Turbo) [5, 47, 48] Minimal medium with glucose: 0.76 h^− 1^ (*E. coli* BL21 (DE3)) [49]
Doubling time	9.4 min to 25.1 min [1, 5, 32, 34]	Up to 20 min under optimal conditions (strain dependent, 27.2 min for *E. coli* Turbo) [5, 47, 48]
Ribosomes in the exponential phase	115,000 for µ = 4 h^− 1^ [31]	70,000 for µ = 2 h^− 1^ [31]
Growth requirements	Moderately halophilic and requires added salt (0.75 – 3% (w/v) NaCl) [28]	Non-halophilic and grows in standard LB-media
Transformation	Linear and plasmid DNA by natural transformation: 10^5^ − 10^6^ CFU µg^− 1^ [50] Plasmids by chemical and electroporation approaches: 10^5^ − 10^6^ CFU µg^− 1^ [5]	No natural transformation Plasmids by chemical and electroporation approaches: 10^6^ − 10^9^ CFU µg^− 1^
Efficiently utilized carbon sources	i.a. glucose, sucrose, maltose, arabinose, glycerol, fructose, starch [7, 34]	i.a. glucose, maltose, lactose, arabinose, glycerol, fructose [51]

* CFU* colony forming units

## Recombinant protein production in *V. natriegens*

Recombinant protein production in bacterial hosts is often limited by poor growth due to metabolic burden or target protein-specific toxicity [52, 53]. Protein misfolding can lead to aggregation into inclusion bodies (IBs), hindering or complicating protein purification. Effective production depends therefore on choosing an appropriate production host as well as ensuring that the production organisms are not subject to limitations, for example due to insufficient oxygen supply or suboptimal media composition. When using a plasmid-based expression, particular attention should be paid to its origin of replication, promoter, selection markers and available tags, e.g. for affinity purification or enhanced solubility of the target protein [54, 55]. If the yield of soluble expressed protein is low, additional strategies may be employed. These include modulation of production rates through variation of expression temperature and inducer concentration, co-expression of chaperones to support correct folding, targeted production as IBs followed by solubilization and refolding, the use of autoinduction media, secretion into the periplasm or culture medium, or cell-free approaches [43, 54–57].

**Fig. 1:**
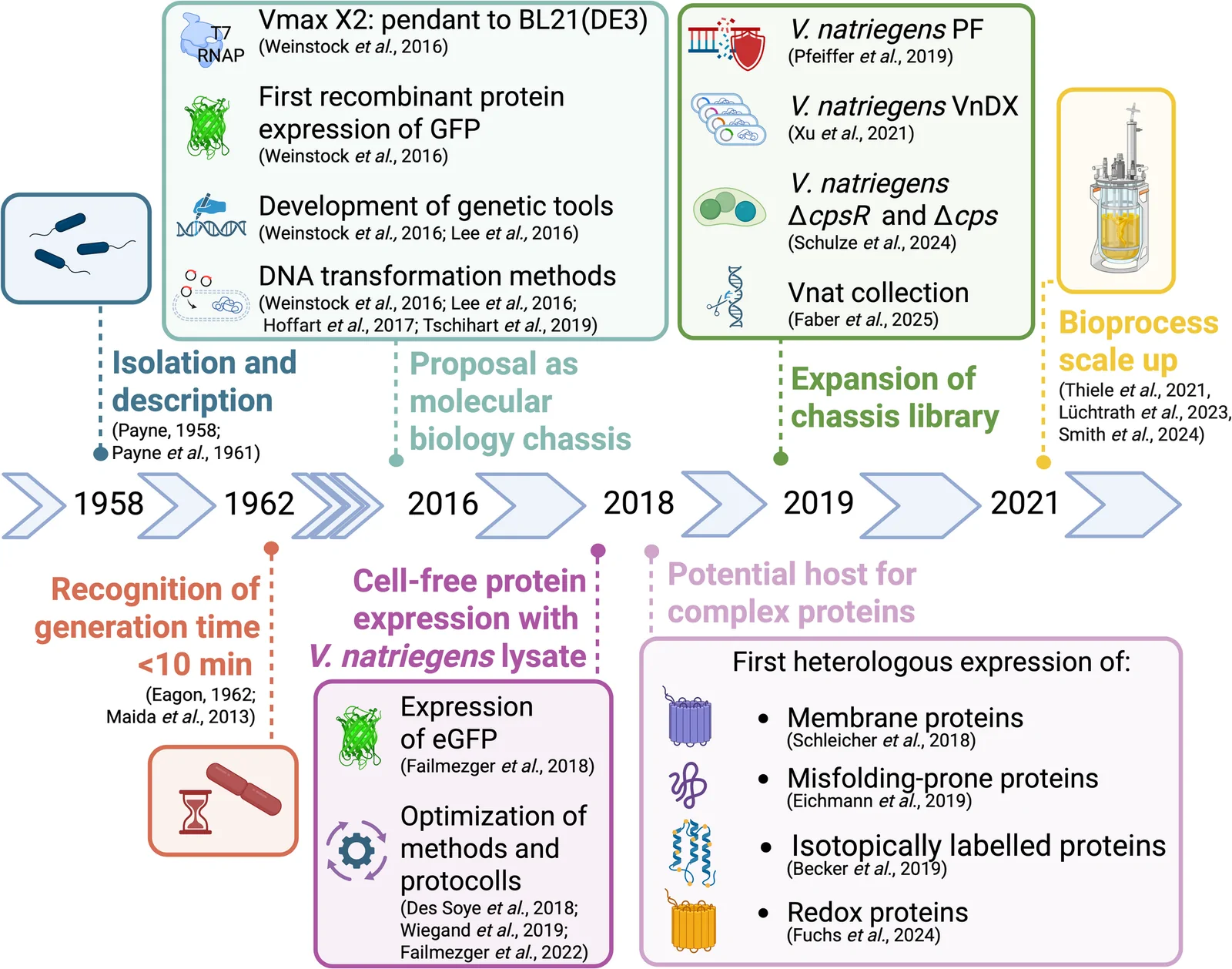
Overview over key achievements regarding the recombinant production of proteins in *V. natriegens*. Figure created with BioRender. *RNAP* RNA polymerase

In the following, some of these approaches will be evaluated in the context of *V. natriegens*, whose protein production and folding characteristics may differ from those of the otherwise similar production host *E. coli* [22], while highlighting potential advantages and limitations of this emerging organism and looking at the question, if faster is always better. In this context, the key achievements over the years for recombinant protein production in *V. natriegens* are summarized in Fig. 1.

### Cellular characteristics of *V. natriegens* as recombinant production host

*V. natriegens* stands out in several distinctive characteristics that facilitate enhanced protein production on a cellular level. Its short doubling time allows substantial shortening of cultivation and process times [30]. Concurrently, with up to 115,000 ribosomes at a growth rate of 4 h^− 1^, *V. natriegens* harbors approximately 30 – 60% more ribosomes than *E. coli*, supporting accelerated biomass formation and potentially increased rates of protein biosynthesis [21, 31] (Table 1). Beyond this, the genetic accessibility of *V. natriegens* is equally important for its applicability as production host. In contrast to *E. coli*, *V. natriegens* exhibits the advantage of natural competence. Strains expressing the master regulator Tfox can take up linear and plasmid DNA without the need for specialized transformation procedures [13, 50, 58]. Specht *et al.* [50] developed a simple transformation protocol for plasmid DNA yielding an efficiency of 10^5 ^− 10^6^ CFU µg^− 1^. Besides natural transformation, plasmids can also be transformed into *V. natriegens* by standard procedures such as conjugation, chemical transformation or electroporation [5, 34]. In this context, reported transformation efficiencies (10^5 ^– 10^6^ CFU µg^− 1^) are substantially lower than those routinely achieved with *E. coli* cloning strains (Table 1) [5]. The genetic and physiological characteristics directly influence the design of expression workflows and bioprocesses with *V. natriegens*. The absence of genes responsible for lactose import and catabolism (*lacY* and *lacZ*) in *Vibrio* species [22, 41, 42] precludes the use of the well-established autoinduction media. Due to their reduced labor requirements and improved mitigation of metabolic burden during induction of gene expression, these media are normally widely used for bacterial recombinant protein production processes [43].

A crucial factor for recombinant protein production is the selection of a suitable promoter. Several inducible promoter systems have been successfully implemented in *V. natriegens*, including isopropyl-β-D-thiogalactopyranosid (IPTG)-, arabinose- and temperature-inducible systems. For example, Weinstock *et al*. demonstrated the functional use of three additional inducible promoters with incorporation of their respective regulators in *V. natriegens*, including the IPTG-inducible P_lacUV5_ and P_trc_ (regulated by *lacI*), the arabinose-inducible P_araBAD_ (regulated by *araC*), and the temperature-dependent P_λpR_ (regulated by *cI857*) [5]. The tetracycline inducible promoter P_tet_ (regulated by *tetR*) and, with a lower response, the light-inducible P_FixK2_ of the pDAWN system can also be employed [59]. Sun *et al*. compared various inducible promoters, showing that higher transcription levels can be achieved with P_tet_ and especially P_trc_ than with P_lacUV5_, although this does not necessarily result in improved soluble protein production [60]. All of the regulatory elements described here were originally established in *E. coli* and only subsequently adapted for use in *V. natriegens*. In addition to inducible systems, constitutive promoters have also been investigated, including members of the Anderson promoter library [61], which show a broad range of expression strengths for recombinant GFP production and exhibit similar trends to those observed in *E. coli* [59]. Furthermore, native promoters such as the P1 promoter described by Tschirhart *et al.* have been shown to provide alternative options for strong gene expression, outperforming commonly used synthetic constitutive promoters [59]. A comprehensive overview of the necessary genetic components for gene expression in *V. natriegens*, such as promoters, ribosome binding sites, terminators, ORIs, and markers, can be found in the review by Bao *et al.* [26]. Ultimately, despite these developments, the repertoire of well-characterized and reliable promoters in *V. natriegens* remains rather limited compared to what can be used in *E. coli* (see e.g. [55]).

The fast growth of *V. natriegens* allows recombinant protein production in shake flasks to be achieved within just a few hours upon induction at temperatures between 30 and 37 °C [5, 15, 22]. However, since most studies on protein production in *V. natriegens* have only been conducted in shake flasks, there is a lack of performance data from bioreactor studies and industrial-scale processes, potentially obscuring challenges of scale-up like oxygen transfer limitations [22, 32]. This is particularly relevant given the high metabolic activity of *V. natriegens*, which is associated with significantly elevated oxygen demand. Reported oxygen uptake rates are approximately 28 mmol g^− 1^ h^− 1^ for *V. natriegens*, compared to 12–14 mmol g^− 1^ h^− 1^ for *E. coli*, indicating more than a twofold higher oxygen requirement [62, 63]. In large scale stirred tank reactors, oxygen transfer rates are frequently a limiting factor, which is why such elevated oxygen demands may therefore exceed the achievable oxygen transfer capacity. Consequently, maintaining sufficient oxygen supply may require increased aeration, higher stirring speeds, optimized impeller configuration or the use of oxygen-enriched air, potentially increasing process complexity and cost [64]. However, in a recent study, the effect of oxygen supplementation on protein production in bioreactor cultivations on a 2 L-scale was investigated. While increased oxygen levels expectedly increased biomass production of *V. natriegens*, no substantial enhancement in protein yield compared to shake flask cultures was observed suggesting a reduced amount of protein per cell [22]. Taken together, these observations indicate that while the high metabolic activity of *V. natriegens* can support rapid biomass formation and potentially high protein productivities, they may also impose constraints on oxygen supply during scale-up, which need to be carefully considered for industrial bioprocess design [44, 45].

In contrast, the production of recombinant proteins in *E. coli* benefits from a high degree of process robustness. Decades of process development have resulted in well-established conditions, such as commonly applied cultivation and induction strategies, which often result in acceptable expression levels without extensive, target-specific optimization. While these workflows provide a robust starting point in *E. coli*, they can be limited in *V. natriegens* since key process parameters, such as induction timing, feeding strategies and metabolic stress, can be affected by the organisms’ differing physiological characteristics. Additionally, cultivation and recombinant protein production in *V. natriegens* have predominantly been investigated in target-specific studies, resulting in limited validation and generalization. Consequently, process development for *V. natriegens* remains largely dependent on the target protein, and established workflows may require substantial re-optimization.

### Genetically modified *V. natriegens* strains for protein production

**Fig. 2:**
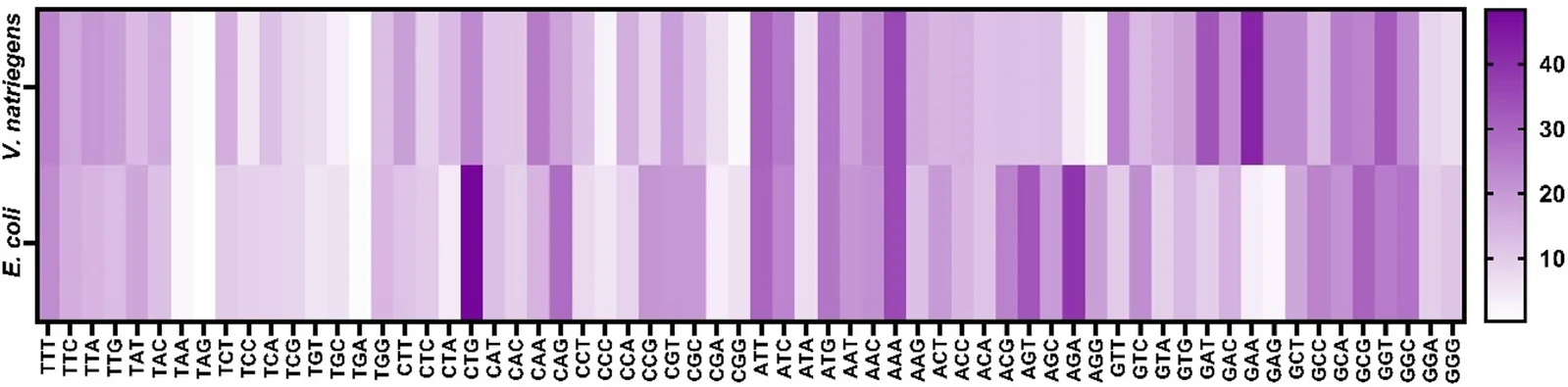
Visualization of codon usage in *E. coli* and *V. natriegens* ATCC 14048 (DSM 759) as frequency per 1000 codons. Data from [69–71]. *TAA*,* TAG and TGA* stop-codons

A current bottleneck of using *V. natriegens* for the recombinant production of complex proteins is the lack of adapted strains. A major development in this direction is the *V. natriegens* Vmax X2 strain [5], which became commercially available in 2017 [65] (Fig. 1). It is designed as a pendant to *E. coli* BL21 (DE3), which encodes a chromosomal T7 RNA polymerase, allowing the use of pET-vectors and IPTG induction [15]. Furthermore, it contains a deletion of the *dns* gene encoding a deoxyribonuclease, a modification analogous to the deletion of *endA1* in *E. coli*, resulting in improved plasmid stability and potentially improved transformation efficiencies [66]. The development of other strains, such as VnDx, were based on Vmax X2. VnDx carries the T7 RNA polymerase in the *dns* locus and exhibits comparable performance in terms of protein production [15]. Notably, the genome of *V. natriegens* harbors more than 100 genes encoding proteases and peptidases [9], among them the lon protease which is inactivated in *E. coli* BL21 [67]. Therefore, a thorough investigation of the proteolytic machinery of *V. natriegens* is essential to develop tailored strains for protein production. Although pET-based vectors are often cited as being directly transferable from *E. coli* to *V. natriegens* Vmax X2, presenting a potential time- and cost-saving advantage, there are limitations to this approach as differences in codon usage between the two species often necessitate codon optimization [10, 68]. As shown in Fig. 2, codon usage in *V. natriegens* and *E. coli* differs significantly. While some codons are used at comparable frequencies (e.g. ATT, AAA, GGA, ACC), particularly rare codons in *V. natriegens* (codon usage < 5, e.g. AGG, CGG, CCC, TGC, AGA) as well as frequent codons (codon usage > 30, e.g. GAT, GAA, GGT) are used substantially more or less frequently in *E. coli*, and vice versa [69, 70]. Therefore, without previous codon optimization, the efficiency of recombinant protein production can be compromised, which partially outweighs the supposed benefits of direct vector transfer.

In addition to the strains described before, Pfeifer *et al.* generated a more robust wildtype variant, called *V. natriegens* PF [72], which has been demonstrated to be a suitable host for recombinant protein production [56]. The deletion of two prophage regions, called *vnp1* and *vnp2*, reduced the strain’s proviral load. This, in turn, made it less susceptible to DNA-damaging or hypo-osmotic conditions. The prophage-free variant PF outperformed the wildtype in competitive growth experiments under hypo-, normal and hyper-osmotic conditions [72]. Recently, Schulze *et al.* [73] showed that *V. natriegens* produces exopolysaccharides which led to significantly increased viscosity and limited oxygen transfer in fed-batch fermentations. Deletion of the transcriptional regulator *cpsR* or of the *cps* cluster resulted in overall low viscosity. Thus, deletion of the prophage regions and *cpsR* or the *cps* cluster can be regarded as a suitable starting point for further strain development with broad applications. Although the development of the modified *V. natriegens* strains described above has overcome some limitations of the wildtype, the limited availability of highly specialized production strains remains a substantial limitation. Accordingly, recent efforts have focused on *V. natriegens* genetic toolkits that enable precise and streamlined strain engineering. Standardized genetic part libraries, such as the Vnat Collection [12] and the Marburg Collection [74] provide valuable resources for the rational design of plasmids and strains for recombinant protein production. The Marburg collection represents a Golden Gate-based toolbox of 191 standardized genetic parts, including promoters, ribosome binding sites, origin of replication, selection markers and terminators, which can be combinatorically assembled for flexible and high-throughput plasmid construction [74]. The Vnat Collection extends the Marburg Collection by introducing orthogonal inducible promoters and a streamlined strategy for operon assembly, thereby improving the flexibility of Golden Gate-based cloning in *V. natriegens* [12]. Beyond plasmid-based approaches recent advances have significantly improved the feasibility of genomic integration in *V. natriegens*. In particular, the development of natural competence-based approaches enabled efficient chromosomal engineering, such as multiplex genome editing by natural transformation (MuGENT), which allows the simultaneous introduction of multiple genomic edits within a single transformation step [58, 75]. Complementary to these approaches, classical allelic exchange strategies, frequently combined with λ-Red recombination systems, enable precise chromosomal modifications [5, 76]. In addition, Clustered Regularly Interspaced Short Palindromic Repeats (CRISPR)/Cas9-based approaches combined with natural transformation have further expanded the genetic toolbox, enabling highly efficient, targeted insertions, deletions and mutations [13]. More recently, CRISPR-associated transposase (CAST)-based systems have further enabled multiplex genome integration and can be used for the genomic introduction of *loxP*-sites, enabling Chromosome Rearrangement and Modification by LoxP-mediated Evolution (CRaMbLE) for identification and modification of functionally relevant genomic regions [77]. Together, these methods allow rational production strain engineering as well as stable chromosomal integration of heterologous genes, which can be particularly beneficial for recombinant protein production, enhancing genetic stability and eliminating the need for antibiotic selection [78, 79]. Although genome engineering in *V. natriegens* is not yet as standardized as in *E. coli*, the available tools already support precise and versatile strain development strategies. A more comprehensive and detailed overview of these tools can be found in Hädrich *et al*. [8].

Beyond the aspects of strain selection shown above, regulatory aspects further influence host selection. Meanwhile, *V. natriegens* has been recommended for inclusion in the European Food Safety Authority (EFSA) Qualified Presumption of Safety (QPS) list for use as a production organism [80]. However, despite this positive regulatory status, practical barriers remain, most notably the limited commercial availability of standardized strains, as exemplified by the commercial discontinuation of the Vmax X2 strain [81], which complicates its broader establishment as a production host.

### Overview of protein production campaigns in *V. natriegens*

Several studies demonstrated promising performance of *V. natriegens* for a broad range of recombinantly produced proteins. For instance, in the work of Xue *et al*., expression conditions of a glucose dehydrogenase as a reporter protein for their pET-compatible VnDX strain were optimized leading to a “what to try first”-protocol. For protein production in shake flasks, it can be broken down to: use (i) Terrific Broth supplemented with v2-salts (TBv2) as medium with a (ii) 10 h long IPTG-induction at (iii) 28 °C [15] (Table 2).

**Table 2:** Composition of typically used media and salt supplementation for *V. natriegens* cultivations

Medium	Composition
LBv2 medium	10 g L^− 1^ tryptone, 5 g L^− 1^ yeast extract, v2-salts^a^ [83]
TBv2 medium	12 g L^− 1^ tryptone, 24 g L^− 1^ yeast extract, 0.5% v/v glycerol, 2.31 g L^− 1^ KH_2_PO_4_, 16.43 g L^− 1^ K_2_HPO_4_·3 H_2_O, v2-salts^a^ [15]
BHIv2 medium	Commonly supplied as a commercially prepared mixture (e.g. Brain Heart Infusion Broth, B9500, Teknova), v2-salts^a^ [5]
v2-salts	204 mM NaCl, 4.2 mM KCl, 23.14 mM MgCl_2_ [5]

^a^Alternatively to supplementation with v2-salts, the total salt concentration can be adjusted to 1.5–3% (w/v) NaCl to support optimal growth of *V. natriegens* [5, 32, 34, 82]

Among 196 genes of interest encoding for commonly used biocatalysts from all enzymes classes, such as glucose dehydrogenases, aminotransferases, lactamases and fructose isomerases, 65% obtained soluble expression in VnDX [15]. Further analysis revealed that 102 proteins were expressed at lower levels in *V. natriegens* than in *E. coli*, while 47 did not show detectable overexpression in either host. Notably, 27 target proteins were produced at comparable levels in both organisms and 20 target genes, encoding e.g. for different reductases, dehydrogenases, transaminases and halogenases, showed even higher expression in *V. natriegens* than in *E. coli*. In this study, almost all coding sequences were codon-optimized for *E. coli* constituting a bias against *V. natriegens* [15]. As the cultivation and induction conditions were not optimized for *V. natriegens*, this was a remarkable result and showed promising potential for the efficient production of various proteins [15]. These results can be generalized to other studies as well. The collected production data in this review (Table 3) illustrate even more the highly target-dependent performance of *V. natriegens* compared to *E. coli*.

Overall, *V. natriegens*, predominantly represented by the engineered Vmax X2 strain, has demonstrated the ability to produce a broad range of heterologous proteins at yields comparable to or exceeding those obtained in *E. coli*. Notably, *V. natriegens* partly showed successful protein production, even under circumstances where expression in *E. coli* was not possible. A number of proteins, originating from *Vibrio* species, including cholera toxin (CT) and the N-acetyl-glucosamine binding protein (GbpA) showed up to tenfold improved yields in *V. natriegens* compared to *E. coli* [24]. These results may reflect the closer phylogenetic relationship between host and originating organism, as well as the potential advantage of extracellular accumulation. For downstream processing, protein secretion or protein-assisted release into the cultivation supernatant would be potentially advantageous. Several of the proteins listed were released into the medium by *V. natriegens*, either through secretion using signal peptides (for instance, the PelB signal peptide directs proteins in *V. natriegens* not only to the periplasm, as in *E. coli*, but directly into the culture medium [24]) or through release by membrane destabilization. This extracellular accumulation could potentially reduce downstream processing compared to intracellular accumulation [24, 56, 84]. However, secretion pathways and secretion behavior in *V. natriegens* are still poorly characterized [5]. In addition, while members of the genus *Vibrio* are known to secrete extracellular proteases [85], their presence and activity in *V. natriegens* have not been systematically investigated, and potential proteolytic degradation of secreted recombinant proteins cannot be excluded.

Beyond proteins originally derived from *Vibrio* species, *V. natriegens* outperformed *E. coli* in case of several challenging protein targets. For instance, fungal nematotoxins (CCTX2) and insect derived peptides, which were insoluble or failed to express in *E. coli*, were obtained in the mg mL^− 1^-range in Vmax X2 [24, 68]. To further quantify these observations, reported yields were normalized to expression time in order to approximate volumetric productivities (mg L^− 1^ h^− 1^), enabling comparison across heterogeneous studies. For challenging protein targets, including toxins (CT, LT) as well as structurally demanding proteins such as GbpA and CCTX2, volumetric productivities in *E. coli* (overexpressed in C41/43 (DE3) or BL21 Star (DE3)) range from approximately 0.02 to 0.83 mg L^− 1^ h^− 1^, whereas *V. natriegens* Vmax X2 reaches substantially higher values of 0.33 to 4.09 mg L^− 1^ h^− 1^ under comparable cultivation times [24]. For standard recombinant proteins like eYFP, *V. natriegens* showed moderately higher volumetric productivities compared to *E. coli* (BL21 (DE3): 2.7 mg L^− 1^ h^− 1^; Vmax Express: 3.8 mg L^− 1^ h^− 1^) [86]. For sfGFP variants containing noncanonical amino acids, which require orthogonal translation systems and are typically associated with reduced translation efficiency, *V. natriegens* Vmax X2 could achieve substantially higher productivities (28.8 – 89 mg L^− 1^ h^− 1^) compared to *E. coli* BL21 (DE3) (2.3 – 26.8 mg L^− 1^ h^− 1^) [87]. These data indicate that *V. natriegens* can achieve higher protein yields within similar process durations and would therefore reach equivalent target titers in shorter time frames, reflecting improved space-time yields. However, it should be noted that these comparisons are based on heterogeneous small-scale experiments involving different strains, expression systems and cultivation conditions. Standardized process metrics such as specific productivity are rarely reported for *V. natriegens*, and many studies only provide qualitative or relative expression data. This currently limits cross-study comparisons and highlights the need for more systematic and quantitative bioprocess characterization of this emerging host.

Despite these generally higher productivities, the performance of *V. natriegens* remains strongly protein dependent. As demonstrated in Table 3, the fast growth of *V. natriegens* does not universally enhance recombinant protein production. Several eukaryotic proteins like human growth hormones or alcohol dehydrogenase from *Saccharomyces cerevisiae* remained largely insoluble or poorly expressed in both organisms [88]. For a mycobacterial aminoacylase, recombinant yields were improved using an engineered *E. coli* strain (ArcticExpress (DE3)) at low expression temperatures (12 °C) combined with co-expression of chaperones [41]. However, such conditions are difficult to implement at industrial scale production, suggesting that alternative hosts like *V. natriegens* may offer more practical strategies for large scale recombinant protein production. Overall, the performance of *V. natriegens* in recombinant protein production remains strongly protein-dependent. In light of the rapid progress in artificial intelligence and machine learning together with sequence-based protein structure prediction, an important goal is to enable a priori assessment of which host would be a better choice for a given protein.

### Cell-free protein production using *V. natriegens* lysate

Cell-free protein synthesis (CFPS) provides a versatile environment, that circumvents physiological constraint of in vivo expression systems, enabling the production of such proteins that cannot be produced at sufficient levels in living cells [57]. While CFPS is currently not widely applicable for large-scale industrial production, recent advances demonstrate substantial scalability, with reaction volumes of up to 100 L reported and near-linear scaling behavior [89, 90]. In these systems, protein titers of 1 – 3 g L^− 1^ have been achieved for model proteins such as GFP [91, 92], whereas titers of more complex targets such as cytokines are typically below 1 g L^− 1^ [89, 90]. With its exceptionally high translational capacity in mind, *V. natriegens* lysate-based CFPS systems have high potential to complement conventional in vivo expression [93, 94]. This expectation is primarily based on the high ribosome content of *V. natriegens*, suggesting a highly active translation machinery and consequently potentially increased CFPS productivity [95]. Accordingly, first protocols based on cell lysates of *V. natriegens* have been established (Fig. 1) [94, 95]. By optimizing cultivation and cell lysis conditions as well as reaction components (salts, amino acids, etc.), protein yields comparable to *E. coli*-based CFPS systems were achieved. With different approaches, up to 1.6 g L^− 1^ of various GFP variants have been reported, which is comparable to established *E. coli*-based CFPS systems [91, 93–95]. However, despite the theoretical advantages, current *V. natriegens* CFPS systems therefore do not yet consistently outperform *E. coli* in terms of overall protein yield, indicating that further optimization is required [94]. Notably, strategies that improve CFPS performance in *E. coli*, such as gene knockouts, have not yet resulted in comparable benefits in *V. natriegens*, highlighting the need for organism specific optimization [95]. Nevertheless, *V. natriegens* already offers practical advantages in extract preparation like higher lysate yields (8 – 12 mL per liter of culture compared to 1 – 3 mL for *E. coli*) and reduced sensitivity to the time of harvest, as cells from both exponential and stationary phase can be used [95]. These features enable a faster and more flexible lysate production workflow. Together with its demonstrated robustness, including storage and handling stability enabled by lyophilization and reactivation, this highlights the potential of *V. natriegens* CFPS systems as a complementary platform for protein production [95]. However, further optimization will be required to fully exploit the high ribosome content of *V. natriegens* and translate this into improved CFPS performance.

**Table 3:** Overview of recombinant proteins previously produced in *V. natriegens*, including their original host organisms

Protein	Original host	Production strain	Secretion or assisted release	Protein yield or activity		References
				*E. coli*	*V. natriegens*	
Aminoacylase (MsAA)	*Mycolicibacterium smegmatis*	*E. coli* BL21 (DE3) *E. coli* ArcticExpress *V. natriegens* (Vmax X2)	No	32.7 U mL^− 1^ 139.1 U mL^− 1^	15.5 U mL^− 1^	[41]
Archaeal catalase-peroxidase (AfKatG)	*Archeoglobus fulgidus*	*V. natriegens* PF	Yes^a^	–	117.9 mg L^− 1^	[56]
Archaeal catalase-peroxidase (AfKatG)	*Archaeoglobus fulgidus*	*E. coli* MC4100 *V. natriegens* (Vmax X2)	No	Comparable yields		[88]
Cholera toxin (CT)	*Vibrio cholerae*	*E. coli* OverExpress C43 (DE3) *V. natriegens* (Vmax X2)	Yes	0.2 mg L^− 1^	2–12 mg L^− 1^	[24]
C-type cytochromes proteins	*Acidithiobacillus ferrooxidans* CCM4253	*E. coli* T7 Express *V. natriegens* (Vmax X2)	No	–	–	[96]
Enhanced green fluorescent protein (eGFP)	*Aequorea victoria*	*Vibrio-based* cell-free protein synthesis system	No	–	0.40 g L^− 1^	[94]
Enhanced yellow fluorescent protein (eYFP)	*Aequorea victoria*	*E. coli* BL21 (DE3) *V. natriegens* (Vmax Express)	No	44 mg L^− 1^	62 mg L^− 1^	[86]
Enhanced yellow fluorescent protein (eYFP)^b^	*Aequorea victoria*	*E. coli* BL21 (DE3) *V. natriegens* (Vmax X2)	No	47.5 mg L^− 1^	75 mg L^− 1^	[35]
FK506 binding protein^b^	*Homo sapiens*	*E. coli* BL21 (DE3) *V. natriegens* (Vmax X2)	No	60 mg L^− 1^	302.5 mg L^− 1^	[35]
Fungal nematotoxin (CCTX2)	*Coprinopsis cinerea*	*E. coli* C41 (DE3) *V. natriegens* (Vmax X2)	No	0.4 mg L^− 1^	10 mg L^− 1^	[24]
Glucose dehydrogenase	*Bacillus subtilis*	*E. coli* BL21 (DE3) *V. natriegens*VnDx	No	123 U mL^− 1^	195 U mL^− 1^	[15]
Heat-labile Enterotoxin (LT)	*E. coli*	*E. coli* *V. natriegens* (Vmax X2)	Yes	0.1 mg L^− 1^	7 mg L^− 1^	[24]
Human growth hormone (hGH)	*Homo sapiens*	*E. coli* BL21 (DE3) *V. natriegens* (Vmax X2)	No	Significantly lower production in Vmax X2		[88]
Insect metalloproteinase inhibitor (IMIPI)		*E. coli Rosetta-gami™ B(DE3)pLysS* *V. natriegens*	Yes	2.2 mg L^− 1^	8.5 mg L^− 1^	[84, 97]
Insect metalloproteinase inhibitor (IMPI)	*Galleria mellonella*	*E. coli* BL21 (DE3) *V. natriegens* (Vmax X2)	No	5 – 6 mg mL^− 1^	insoluble	[67]
Intermembrane phospholipid transport system binding protein (MlaC)	*E. coli*	*E. coli* BL21 (DE3) *V. natriegens* (Vmax X2)	No	Lower yields than with Vmax X2	58 mg L^− 1^	[86]
GTPase KRAS4a KRAS4b	*Homo sapiens*	*V. natriegens* (Vmax X2)	No	–	–	[98]
GTPase KRAS4b	*Homo sapiens*	*E. coli* BL21 (DE3) Star *V. natriegens* (Vmax X2)	No	29 mg L^− 1^	41 mg L^− 1^	[22]
Leaf branch compost cutinase (LCC) variant (LCC_ICCG_)	–	*V. natriegens* (Vmax X2)	No	60 mg L^− 1^	100 mg L^− 1^	[99]
Lucimycin	*Lucilia sericata*	*E. coli* BL21 (DE3) *V. natriegens* (Vmax X2)	No	insoluble	20 mg mL^− 1^	[68]
Manganese-dependent bacterial peroxidase (MDBP)	*–*	*V. natriegens* PF	Yes^a^	–	36.5 mg L^− 1^	[56]
*N*-Acetyl glucosamine-binding protein A (GbpA)	*Vibrio cholerae*	*E. coli* BL21 (DE3) Star *V. natriegens* (Vmax X2)	Yes	15 mg L^− 1^	42 – 90 mg L^− 1^	[24]
PETase	*Ideonella sakaiensis*	*V. natriegens* (Vmax X2)	No	100 mg L^− 1^	175 mg L^− 1^	[99]
PETase and MHETase	*Ideonella sakaiensis*	*V. natriegens* (Vmax X2)	No	–	–	[100, 101]
RAF1 Kinase	*Homo sapiens*	*E. coli* BL21 (DE3) Star *V. natriegens* (Vmax X2)	No	7 mg L^− 1^	51 mg L^− 1^	[22]
RAF1/KRAS	*Homo sapiens*	*V. natriegens* (Vmax X2)	No	–	–	[102]
RBD-1-2G nanobody	–	*E. coli* BL21 (DE3) Star *V. natriegens* (Vmax X2)	No	0.2 mg L^− 1^	2.5 mg L^− 1^	[22]
SARS-CoV-2 nanobodies	*–*	*V. natriegens*	No	–	–	[103]
Superfolder green fluorescent protein (sfGFP)	*Aequorea victoria*	*Vibrio-based* cell-free protein synthesis system	No	–	0.26 g L^− 1^	[93]
Superfolder green fluorescent protein (sfGFP)	*Aequorea victoria*	*Vibrio-based* cell-free protein synthesis system	No	–	1.6 g L^− 1^	[95]
*Taq* polymerase	*Thermus aquaticus*	*V. natriegens* PF	Yes^a^	–	26.5 mg L^− 1^	[56]
Tyrosine phenol lyase (TPL)	*Fusobacterium nucleatum*	*E. coli* BL21 (DE3) *V. natriegens* (Vmax X2)	No	–	–	[25]
Uricase	*Lucilia sericata*	*E. coli* BL21 (DE3) *V. natriegens* (Vmax X2)	No	insoluble	2.5 mg mL^− 1^	[68]
Yeast alcohol dehydrogenase (ADH)	*Saccharomyces cerevisiae*	*E. coli* BL21 (DE3) *V. natriegens* (Vmax X2)	No	No soluble form	No soluble form	[87]
Multiple proteins (*n* = 192)^c^	Various organisms	*E. coli* BL21 (DE3) *V. natriegens* (VnDX)	–	High-throughput expression screening (no quantitative yield data)		[15]

When reported in the literature, production yields in *V. natriegens* and *E. coli* are provided

– No reported quantitative values for this protein could be found in published studies

^a^Extracellular transport was not achieved by secretion, but by reduced membrane integrity

^b^Isotopically labelled variants of these proteins were produced

^c^A total of 196 proteins, representing all seven enzyme classes, were produced in this study without reported yields; for further details, see [15]

### Membrane protein production in *V. natriegens*

Beyond the production of soluble proteins, more complex target classes such as membrane proteins remain particularly challenging for recombinant production. About 30% of naturally occurring proteins are predicted to be embedded in biological membranes, where they play essential roles in biological processes such as cell-cell communication, cellular recognition, metabolism and signaling [104–106]. Consequently, many membrane proteins are important targets in medical and pharmaceutical research. However, their hydrophobic nature and complex folding requirements as well as the dependence of proper membrane insertion and a suitable membrane lipid composition make them challenging to produce recombinantly [107–111]. Despite these challenges, *V. natriegens* has already demonstrated its capacity to produce several functionally active membrane proteins (Table 4).

**Table 4:** Overview of membrane-associated proteins previously produced in *V. natriegens*, including their original host organisms

Membrane-associated protein	Original host	Production strain	References
NADH: quinone oxidoreductase (NQR)	*Vibrio cholerae*	*V. natriegens* ATCC 14048	[23]
Multiple resistance and pH (Mrp) cation/proton antiporter	*Vibrio cholerae*	*V. natriegens* ATCC 14048	[23]
C-type cytochrome CycA	*Acidithiobacillus ferrooxidans*	*V. natriegens* Vmax X2	[96]
C-type cytochrome Cyc2	*Acidithiobacillus ferrooxidans*	*V. natriegens* Vmax X2	[96]

Quantitative production yields or titers were not reported in the original studies; available data are limited to qualitative/functional assays.

Already in 2018, Schleicher *et al.* demonstrated the recombinant production of multi-subunit membrane protein complexes in *V. natriegens* [23]. Remarkably, both the Na⁺-translocating NADH: quinone oxidoreductase (NQR) and the Multiple resistance and pH (Mrp) cation/proton antiporter complex, mediating the exchange of protons for Li^+^, Na^+^, and K^+^ ions, were produced in *V. natriegens* in fully assembled and functional forms with comparable activities (1.6 U mg^− 1^) [23] to those reported for *E.coli* [112], without additional co-expressed components as often necessary for other production hosts [23, 107, 113, 114]. Fuchs *et al*. provided further evidence for the organism’s potential to produce complex membrane proteins [96]. Heterologous production of c-type cytochromes in *E. coli* is hindered, since the *ccmABCDEFGH* genes required for heme-c incorporation are only expressed under anaerobic conditions. Traditionally, this bottleneck has been circumvented by co-expression of the *ccm* gene from the pEC86 vector [96]. In contrast, *V. natriegens* Vmax X2 enabled the recombinant production of several difficult to produce redox proteins from *Acidithiobacillus ferrooxidans* without such co-expression strategies. These redox proteins included CycA, anchored in the inner membrane, as well as the outer-membrane β-barrel cytochrome Cyc2, both produced from T7-based plasmids originally developed for *E. coli*, highlighting the compatibility of established expression tools. In comparison with *E. coli* T7 Express, *V. natriegens* Vmax X2 demonstrated a greater biomass formation (26.6 vs. 18.6 g L^− 1^ cell wet weight) and increased total membrane protein yields (16.4 vs. 9.5 mg mL^− 1^) [96]. Notably, CycA could not be produced in *E. coli* under the tested conditions but was successfully expressed in *V. natriegens.* In addition, higher band intensities of the target cytochrome Cyc2 indicated a greater yield of recombinant protein in *V. natriegens* [96], underlining its potential for producing redox-active membrane proteins more productive compared to *E. coli*.

Beyond in vivo expression approaches, CFPS has been successfully applied for membrane protein production in established systems such as *E. coli*. This is enabled by the flexible reaction environment of CFPS, which allows the incorporation of membrane-mimicking systems such as detergents, liposomes, or nanodiscs to support proper folding and insertion [90, 115–118]. While this approach has not yet been widely implemented for *V. natriegens*, it represents a promising future strategy that may further expand its applicability.

**Fig. 3:**
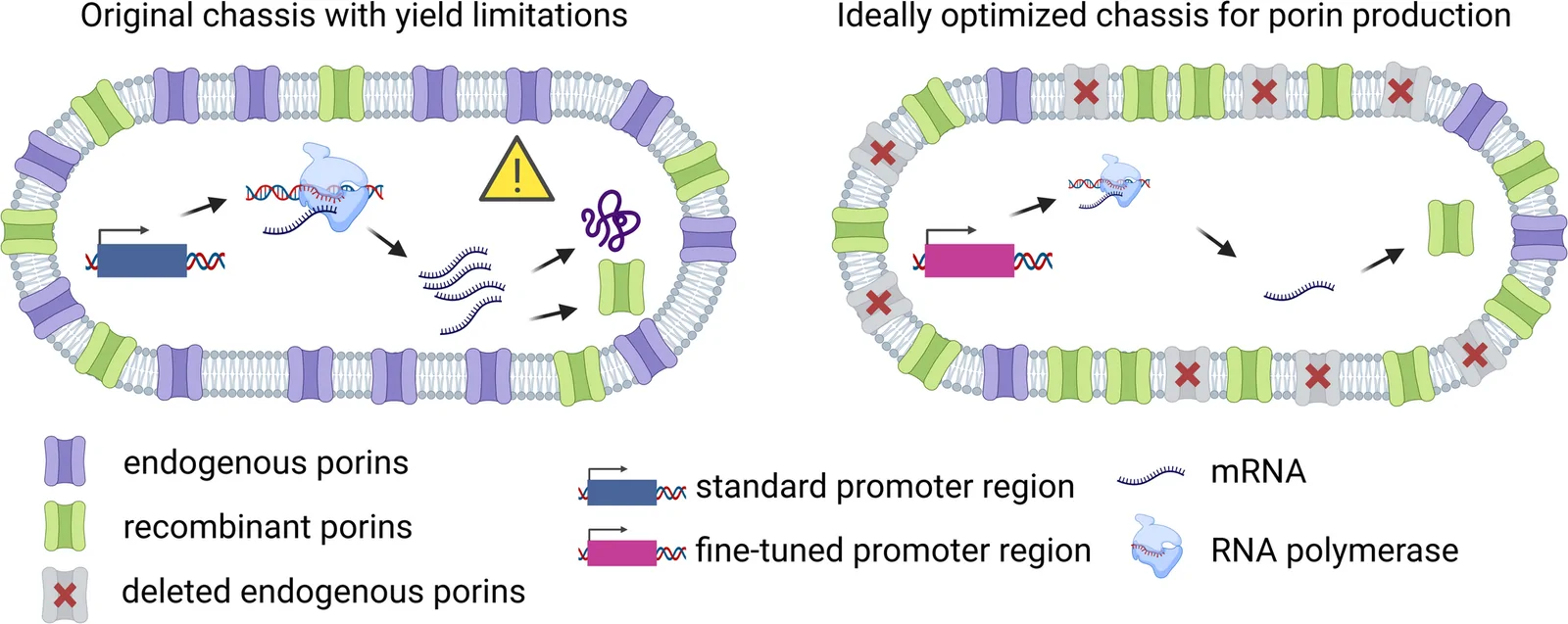
Schematic comparison of the outer membrane of a non-optimized, porin producing *V. natriegens* strain with yield limitations, and of a proposed host design. The latter represents a conceptual wish list, that includes deleting selected membrane porins in the outer membrane and adjusting promoter strength to facilitate recombinant porin production. Figure created with BioRender

In addition to the production of membrane proteins for functional and structural studies, *V. natriegens* also offers opportunities for membrane-associated transport applications. In this context, porins represent particularly attractive targets, as they enable controlled substrate transport in natural or artificial compartment systems [119, 120]. Outer membrane porins (OMPs) can be used for the passive or active transport of small molecules [111], enabling reaction compartments, engineered microbial platforms or artificial molecular communication. However, OMP biosynthesis involves either post-translational or co-translational transport across the inner membrane and insertion via the β-barrel assembly machinery (BAM complex) in the outer membrane [121]. Since this pathway has a finite capacity, overexpression of recombinant OMPs can overcrowd the translocation and periplasmic folding machinery, while additionally endogenous OMPs further limit available space, potentially reducing production yields. In *E. coli*, these limitations have been addressed by reducing the burden of endogenous OMP production. For instance, four major porins, notably OmpF, OmpA, OmpC and LamB, are deleted in the strain *E. coli* BL21 (DE3) Omp8. The mitigated membrane saturation can improve heterologous production of target membrane proteins [122]. Similarly, specialized *E. coli* strains such as C41 (DE3) und C43 (DE3) have been developed to reduce the metabolic stress associated with difficult-to-express proteins, including membrane proteins. In contrast, the fast growth and high translational capacity of *V. natriegens* [9, 31], which is often highlighted as an advantage for recombinant protein production [22], can be a double-edged sword. High rates of transcription and translation can overwhelm folding and membrane insertion pathways, potentially resulting in misfolded proteins, incorrect assembly of complex membrane protein complexes and inefficient membrane integration [123]. Consequently, although *V. natriegens* is highly productive for easy targets, it may be less robust for difficult targets than specialized *E. coli* strains such as C41 (DE3), C43 (DE3) or Lemo21 (DE3), where weaker promoters and tunable t7 RNA polymerase activity, mediated by controlled expression of T7 lysozyme, can reduce cellular stress [124–126]. Currently, comparable specialized *V. natriegens* strains with features such as regulated expression and/or reduced endogenous protein burden are lacking, emphasizing the wish to develop such strains to fully exploit the organism’s potential for recombinant membrane protein production (Fig. 3).

## Conclusions and emerging research directions

The rapid growth and metabolic flexibility of *V. natriegens* clearly position it as a promising addition to the current repertoire of microbial hosts for recombinant protein production. Notably, *V. natriegens* shows also rapid growth under anaerobic conditions [34], which might be a promising trait to produce oxygen sensitive proteins. In laboratory scale applications, these properties enable accelerated strain construction and rapid initial screening of heterologous protein production compared to conventional hosts. However, the translation of these biological characteristics into robust and scalable production processes remains at an early stage. To date, most reported proteins production studies using *V. natriegens* are limited to shake flask cultivations, and systematic investigations into high cell cultivation and feeding strategies in bioreactor approaches are scarce. In particular for industrial implementation, the organism’s high oxygen demand and requirement for elevated salt concentrations raises unsolved questions regarding corrosion of bioreactors as well as downstream compatibility. Consequently, currently *V. natriegens* does not appear to be a direct replacement for *E. coli* in established large-scale protein production processes. Similarly, while *V. natriegens*-based CFPS have been demonstrated and may offer advantages, their relevance for industrial protein production remains limited.

A key limitation of *V. natriegens* is still the comparatively narrow spectrum of engineered chassis strains and standardized tools available for addressing specific expression challenges, compared to *E. coli*, which has benefited from decades of optimization. Numerous specialized *E. coli* strains exist, that target bottlenecks in recombinant protein production. Examples include strains such as BL21-CodonPlus or Rosetta, which enhance tRNA availability to mitigate translation limitations [127], or Nico21 (DE3), which improves the purification of His-tagged proteins by reducing endogenous metal-binding contaminants during immobilized metal affinity chromatography (IMAC) [128]. SHuffle^®^ T7 or Origami™ 2 (DE3) enable efficient cytoplasmic disulfide bond formation and thereby avoid for example cell-free systems [129]. Furthermore, strains such as C41 (DE3) and C43 (DE3) as well as Lemo21 (DE3) provide a reduced or tunable T7 RNA polymerase activity, facilitating the production of challenging proteins, such cytotoxic or membrane-associated ones [124, 126, 130]. Together, these strains illustrate the high degree of specialization for *E. coli* as protein production host and define a benchmark for future strain engineering efforts in *V. natriegens*. The development of comparable specialized chassis strains, tailored for the production of membrane proteins and the improvement of secretion, or equipped with additional genes, such as those that enable lactose metabolism for autoinduction media, comprise a wish list of unlocking capabilities beyond those of the wildtype organism. Rather than serving as a direct replacement, *V. natriegens* potentially functions as a complementary system, broadening the range of available strategies. In practice, the selection of the organism of choice will continue to depend heavily on the requirements of the target protein and the process environment. In this context, the “plug-and-play” option between *E. coli* and *V. natriegens* by compatible plasmids and expression workflows has the potential to accelerate future transformation. Whether *V. natriegens* will in the end evolve from a fast-growing laboratory curiosity into a standardized chassis for industrial protein production is contingent upon significant advancement of genetic tool development, the establishment of specialized strains and, most crucially, the demonstration of robust scalable and economically viable bioprocesses. Until such milestones are reached, *V. natriegens* should not be viewed as substitute for *E. coli*, but as a promising and still developing addition to the established portfolio of microbial protein production hosts. Ultimately, while faster growth can indeed be better, it is not always.

## Data Availability

No datasets were generated or analysed during the current study.
